# Supplementary material for: Chromothripsis during telomere crisis is independent of NHEJ, and consistent with a replicative origin
Source: Genome Res. 2019 May;29(5):737–49. doi: 10.1101/gr.240705.118 (PMC6499312; doi:10.1101/gr.240705.118)
Supplement: Supplemental Material [file supp_gr.240705.118_Supplemental_file_1.zip › contigs/annotated_contigs/DB113/contig.3.DB113_length_588_mean_cov_13.7329931973.docx]

**DB113_length_588_mean_cov_13.7329931973**

```
AATCTTGAAATTTGGAGAAAGACCAAATAAATAAACAGGGCTAGGTGCAATGGCCCACGCCTATAATCCCAGCACTTTGGGAGACTGAC
 >chr10:13269397-13269587 + E=1e-99
ATGGGCAGATTACTTGAGGTCAGGAGTTCGTGACCAGCCTGGCCAACATAGTAAATCCCCATCTCTACCCAAAATACAAAAATTAGCTG

GGCATAGCGGTG|AAACCCTGTCTCTACGAAC|CTGCAAGGCAGGTTCATTCTCATCCTGGTTCCTGTGTCATCTCTGCAGATGTGTTC
                                >chr10:12953101-12953284 + E=2e-95
CCTTGTCACCCCATAAGTCACCTCTCCTAATGAGGCTGTTGCATCACCTGCTTCTCTTCAGTGCCTTATAACATTGTTGGATTTCCTTA

CTAATGGCTAATTTGTTCTTTCTTCCACTAGACTAAAA|TCCCAGGTTTGCGCCATTCTCCTGCCTCAGCCTCCCGAGTAGCTGGGACT
                                        >chr10:12316401-12316597 + E=3e-106
GCAGGTGCCCGCCACCACGCCCAGCTGATTTTTTTGTGTATTTTTTTAGTGGAGATGGGGTTTCACCGTATTAGCCAGGACGATCTCGA

TCTCCTGACCTTGTGATCTGCTCGCCTCGCCCTCCCAAAGTGCTGGGATTACAGGTG
```
